# Carboplatin with Decitabine Therapy, in Recurrent Platinum Resistant Ovarian Cancer, Alters Circulating miRNAs Concentrations: A Pilot Study

**DOI:** 10.1371/journal.pone.0141279

**Published:** 2015-10-20

**Authors:** Eric A. Benson, Todd C. Skaar, Yunlong Liu, Kenneth P. Nephew, Daniela Matei

**Affiliations:** 1 Department of Medicine, Indiana University School of Medicine, Indianapolis, Indiana, United States of America; 2 Division of Clinical Pharmacology, Indiana University School of Medicine, Indianapolis, Indiana, United States of America; 3 VA Roudebush Hospital, Indiana University School of Medicine, Indianapolis, Indiana, United States of America; 4 Department of Medical and Molecular Genetics, Indiana University School of Medicine, Indianapolis, IN, United States of America; 5 Center for Computational Biology and Bioinformatics, Indiana University School of Medicine, Indianapolis, Indiana, United States of America; 6 Departments of Cellular and Integrative Physiology, Indiana University School of Medicine, Indianapolis, Indiana, United States of America; 7 Department of Obstetrics and Gynecology Medical Sciences Program Indiana University School of Medicine, Indianapolis, Indiana, United States of America; 8 Indiana University Melvin and Bren Simon Cancer Center, Indianapolis, Indiana, United States of America; 9 Department of Biochemistry and Molecular Biology, Indiana University School of Medicine, Indianapolis, Indiana, United States of America; SAINT LOUIS UNIVERSITY, UNITED STATES

## Abstract

**Objective:**

Plasma miRNAs represent potential minimally invasive biomarkers to monitor and predict outcomes from chemotherapy. The primary goal of the current study—consisting of patients with recurrent, platinum-resistant ovarian cancer—was to identify the changes in circulating miRNA concentrations associated with decitabine followed by carboplatin chemotherapy treatment. A secondary goal was to associate clinical response with changes in circulating miRNA concentration.

**Methods:**

We measured miRNA concentrations in plasma samples from 14 patients with platinum-resistant, recurrent ovarian cancer enrolled in a phase II clinical trial that were treated with a low dose of the hypomethylating agent (HMA) decitabine for 5 days followed by carboplatin on day 8. The primary endpoint was to determine chemotherapy-associated changes in plasma miRNA concentrations. The secondary endpoint was to correlate miRNA changes with clinical response as measured by progression free survival (PFS).

**Results:**

Seventy-eight miRNA plasma concentrations were measured at baseline (before treatment) and at the end of the first cycle of treatment (day 29). Of these, 10 miRNAs (miR-193a-5p, miR-375, miR-339-3p, miR-340-5p, miR-532-3p, miR-133a-3p, miR-25-3p, miR-10a-5p, miR-616-5p, and miR-148b-5p) displayed fold changes in concentration ranging from -2.9 to 4 (p<0.05), in recurrent platinum resistant ovarian cancer patients, that were associated with response to decitabine followed by carboplatin chemotherapy. Furthermore, lower concentrations of miR-148b-5p after this chemotherapy regimen were associated (P<0.05) with the PFS.

**Conclusions:**

This is the first report demonstrating altered circulating miRNA concentrations following a combination platinum plus HMA chemotherapy regiment. In addition, circulating miR-148b-5p concentrations were associated with PFS and may represent a novel biomarker of therapeutic response, with this chemotherapy regimen, in women with recurrent, drug-resistant ovarian cancer.

## Introduction

Each year, over 200,000 women are diagnosed with ovarian cancer worldwide; more than 21,000 of those are American. Approximately 15,000 American women die from epithelial ovarian cancer (EOC) every year; of the various types of ovarian cancer, high grade serous ovarian cancer is the most common (HGSOC) [1, 2]. For HGSOC identification, a well-established serum biomarker is the cancer antigen 125 (CA-125). Although CA-125 identifies most late stage primary and relapsed disease and largely correlates with disease burden, CA-125, in terms of sensitivity and specificity, is not predictive of the risk of relapse or chemotherapeutic response [2, 3]. In general, EOC biomarkers are needed, and circulating miRNAs, which have associated with disease outcome [4], may represent a novel source of clinically informative ovarian cancer biomarkers.

MicroRNAs (miRNAs) are short RNAs differentially expressed in many disease states, including cancer. They alter the target protein synthesis by regulating post-transcriptional gene expression [5–7]. miRNAs are released into the plasma by tissues and tumors [6, 8]. miRNAs are relatively heat stable in both whole blood and plasma [9, 10]. Circulating miRNAs have been associated with early detection and prognosis of EOC [11, 12] and may predict therapeutic response.

First-line EOC treatments utilize platinum based chemotherapies. These chemotherapies have been shown to change, within tissues, miRNA expression [13–16]. In this study, plasma miRNA concentrations were measured from a previously completed phase II clinical trial [17]. In this trial, 17 subjects with platinum resistant recurrent ovarian cancer were tested for efficacy and tolerability of a combination regimen of low dose decitabine (5-aza-2’-deoxycytidine; a DNA methyltransferase inhibitor) and carboplatin. The main objective of the clinical trial was to test the hypothesis that treatment with decitabine would resensitize ovarian tumors to carboplatin. The trial was based on previous preclinical studies indicating that reversal of DNA hypermethylation induces re-expression of tumor suppressor and differentiation associated genes that would restore sensitivity to platinum [18]. The results of the trial demonstrated prolonged progression free survival (PFS of ~10 months) and a promising rate of objective response rates of 35% [17]. Taking advantage of the clinically annotated plasma samples collected for this trial, we determined in response to decitabine followed by carboplatin chemotherapy the changes in circulating miRNAs concentrations. Secondarily, miRNA changes were associated with clinical response.

## Methods

### Patient population

The open label phase II clinical trial and response rates were previously described by Matei et al [17]. Briefly, all patients had platinum resistant EOC and all but one patient had measurable disease as defined by Response Evaluation Criteria in Solid Tumor (RECIST). The patient with non-measureable disease had a CA-125 greater than twice the upper limit of normal and ascites. All patients in this study were older than 18 years, had greater than 3 months life expectancy, and had an Eastern Cooperative Group performance status of 0 or 1. Patients could have received unlimited number of prior cytotoxic therapies; however, they were excluded if they had a prior history of brain metastases, an allergy to carboplatin, uncontrolled medical issues, or grade 2 or higher neuropathy. All patients signed informed consent, and the protocol was approved by the Indiana University Institutional Review Board.

### Treatment plan

Each chemotherapy cycle lasted 4 weeks and consisted of decitabine (Eisai, Tokyo, Japan) given intravenously at 10 mg/m^2^ daily for 5 days; followed by, carboplatin (Bristol Meyers Squibb, New York, New York) given intravenously at an AUC5 on day 8 [17]. Additionally, peg-filgastrim (Amgen, Thousand Oaks, CA) was given on day 9 to prevent prolonged myelosuppression. Treatment was continued until disease progression or intolerable toxicity.

### RNA extraction from plasma

miRNAs were extracted from the plasma following a previously published protocol [9]. Briefly, plasma samples were thawed on ice to ~4°C, and centrifuged at 1500 RCF, 4°C for 10 minutes. Fifty μL of plasma was extracted following the Qiagen miRNeasy plasma protocol (Qiagen, Valencia, CA). Following the Qiazol lysis step, C. elegans miR-39 miRNA (cel-miR-39) was added (5 μL of 5 nM stock) as an internal control for extraction efficiency and normalization. Additionally, 0.3 μL of a stock (0.8 μg/μL) Bacteriophage M2 RNA was added per 200 μL Qiazol buffer to increase miRNA yield. miRNAs were eluted from the purification column twice in 30 μL RNase/DNase free water (total 60 μL) and stored at -80°C.

### Statistical analysis

In this pilot study, the primary outcome was the analysis of the difference in plasma miRNA concentrations between the before and after chemotherapy treatment. All values were normalized to the cel-miR-39 spiked in control. Statistical significance (p-value) in the comparisons of groups was determined by paired T-test. In Table 1, the baseline comparison was two tailed T-test. The heatmaps were completed in Partek software using the delta delta C_T_ (ddC_T_) values. The following parameters were used for the heatmap: no scale; the distance vector = Spearman rank correlation; and the clustering = average. Of note, 1 is no fold-change, while 2 is a doubling of fold change.

**Table 1:** Chemotherapy-induced changes in circulating miRNAs concentrations.

	All subjects Treatment/Baseline^1^		Treatment comparison non-responsive subjects^2^		Treatment comparison in responsive subjects^3^		After treatment comparison responsive/non-responsive^4^	
miRNA	Fold change	p value^a^	Fold change	p value^a^	Fold change	p value^a^	Fold difference	p value^b^
hsa-miR-193a-5p	**-1.70** *	0.033	-1.97	0.058	-1.40	0.37	1.36	0.58
hsa-miR-375	**-1.97** *	0.039	**-2.14** *	0.043	-1.76	0.38	1.03	0.97
hsa-miR-339-3p	**1.92** *	0.049	1.09	0.80	**4.05** *	0.012	2.05	0.22
hsa-miR-340-5p	1.97	0.079	1.52	0.47	**2.79** *	0.048	1.35	0.69
hsa-miR-148b-5p	-1.62	0.15	**-2.86** *	0.0495	1.33	0.16	**3.23** *	0.046
hsa-miR-133a-3p	1.49	0.19	1.25	0.66	**1.88** *	0.015	-1.13	0.89
hsa-miR-25-3p	-1.22	0.40	**-1.82** *	0.042	1.40	0.339	1.01	0.99
hsa-miR-10a-5p	1.25	0.47	-1.14	0.79	**2.00** *	0.024	1.40	0.47
hsa-miR-616-5p	-1.07	0.67	-1.43	0.094	1.38	0.116	**1.97** *	0.049
hsa-miR-532-3p	-1.11	0.70	-1.72	0.11	1.62	0.252	**2.84** *	0.044

^1^The change plasma miRNA concentrations from baseline to after treatment with decitabine and carboplatin.

^2,3^Subgroup analysis of non-responsive subjects (progressed before 7 cycles of therapy) and responsive subjects (did not progress before 7 cycles of therapy), respectively.

^4^Plasma miRNA concentrations are fold differences between responders and non-responders using the after chemotherapy treatment miRNA concentrations. Non-responsive subjects are those that had < 6 months of treatment prior to progression of ovarian cancer; Responsive subjects are those that had > 6 months prior to progression of ovarian cancer based on RECIST.

*p<0.05

^a^Paired T-test

^b^T-test.

### Kaplan Meier curve

Progression free survival (PFS) analysis was conducted with “survival” package in R. Samples were divided into 2 groups by the median value of the “–delta delta CT” values. The log-rank test was performed in R to assess the association between miRNA concentration (high vs. low) with the time to progression, and p-value less than 0.05 was considered significant. Kaplan-Meier curves were used to visually examine the cumulative probability of remaining progression free over time for miRNA sub-groups.

## Results

### Plasma sample selection

**Fig 1:**
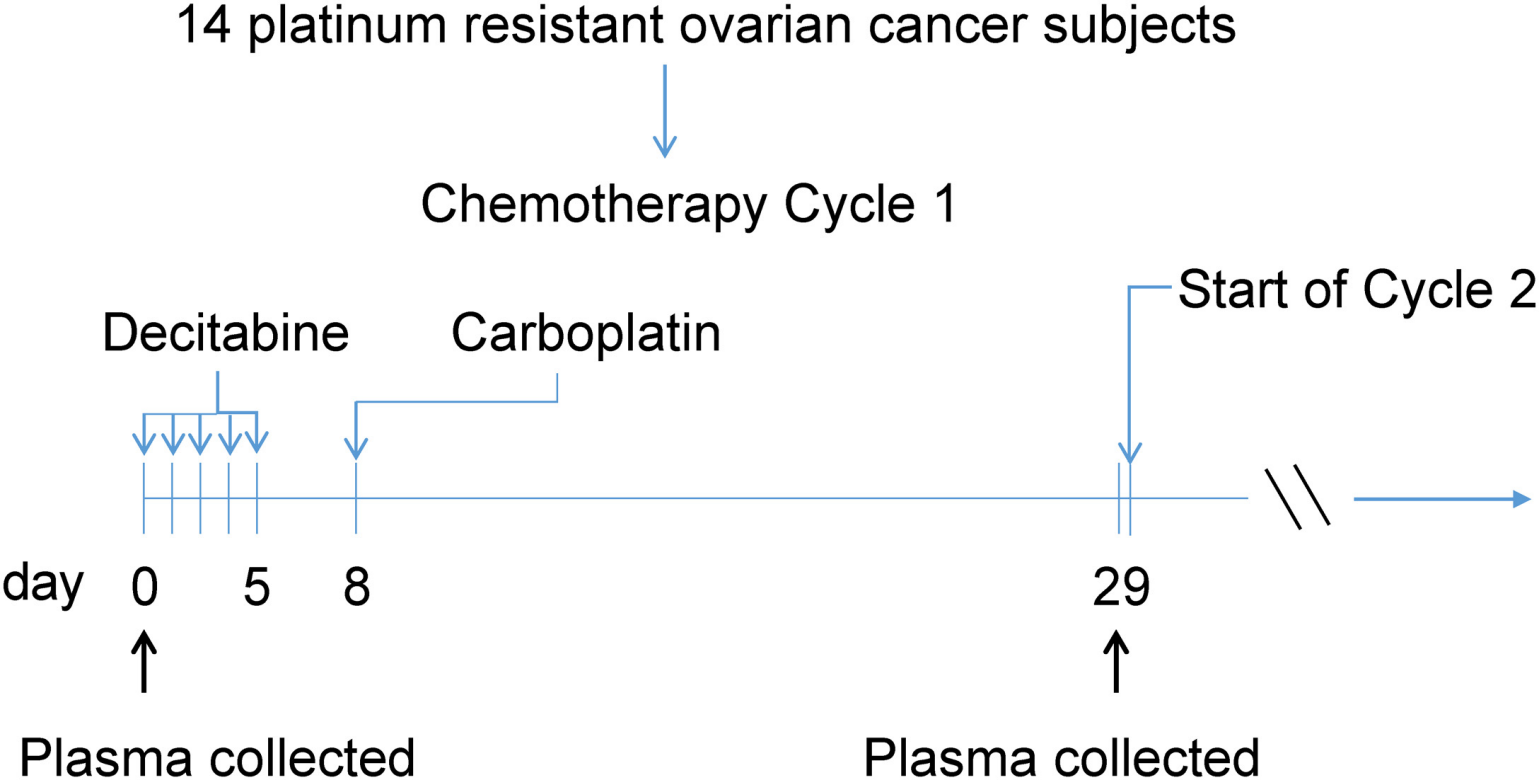
Study Design: Platinum resistant ovarian cancer subjects treated with decitabine and carboplatin. Fourteen platinum resistant ovarian cancer patients were treated with decitabine/carboplatin in a phase II clinical trial. For the first chemotherapy cycle, decitabine was given daily for 5 days; followed by carboplatin given on day 8. miRNA analysis used plasma samples collected at baseline (prior to start of cycle one treatment) and at day 29 (prior to start of second cycle).

Briefly, whole blood was collected in EDTA containing tubes, and then centrifuged to plasma. Samples used for microRNA analysis were collected at two time points: at baseline (prior to the start of the first cycle of treatment); and on day 29 after treatment initiation (post the first cycle of chemotherapy; Fig 1). Samples from 14 of the 17 patients were available for this miRNA analysis. The remaining 3 samples were not analyzed because of insufficient plasma. Samples were analyzed after the first cycle to allow for early miRNA analysis, which may provide evidence toward their future use in early clinical decision-making.

### miRNA selection and concentration

Ninety-three miRNAs were selected for measurement in the 28 samples (14 baseline and 14 after treatment). To select the miRNAs, real-time qPCR miRNA OpenArrays (Life Technologies, Carlsbad, CA), which measure 756 miRNAs, were used to analyze a pooled baseline sample (14 samples) versus a pooled day 29 sample (14 after treatment samples). RNA was extracted from each individual sample and pooled. The miRNAs that were easily quantifiable and with the largest difference between the baseline and day 29 were selected for further analysis of individual samples. The 93 miRNAs selected were analyzed by a custom real-time PCR Taqman miRNA low density array (TLDA) (Life Technologies). For final analysis, of the original 93 miRNAs, 78 had acceptable qPCR curves. Plasma samples were analyzed at each of the two time points (baseline and day 29; 14 patients each), and the same TLDA card was used for both samples. Data are expressed as relative concentrations based on the C_T_ values that have been normalized to the *C*. *elegans* miRNA (5 μL of 5 nM) spiked into the plasma as an internal control.

### Patient characteristics

**Fig 2:**
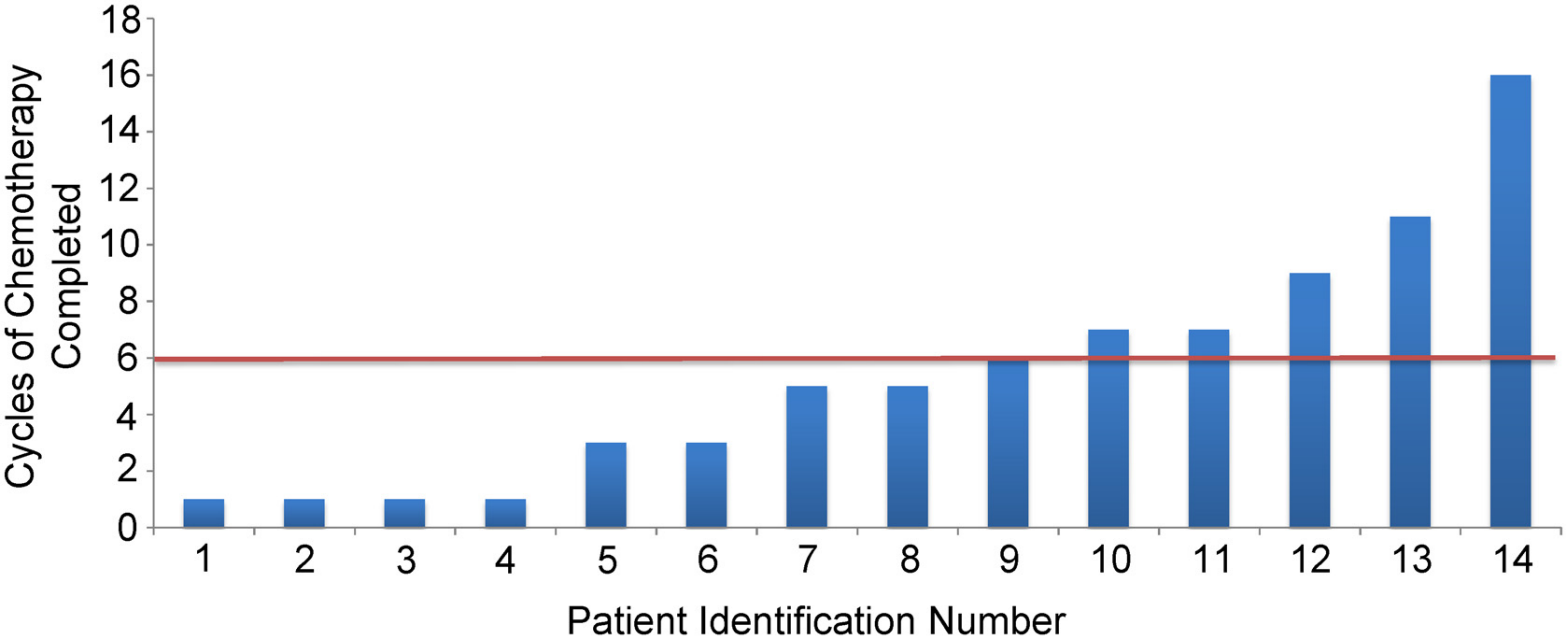
Number of cycles of chemotherapy completed by each subject. The patients’ are numbered and ordered based on the number of received cycles of chemotherapy. Patients were separated by a red line to distinguish chemotherapy responders and non-responders. Responders remained free of disease progression for at least 6 cycles (n = 6); and non-responders remained free of disease progression less than 6 cycles (n = 8). Each cycle was 28 days long.

Fourteen of the original 17 patients in the parent trial had plasma samples available for miRNA analysis. Disease status was assessed using RECIST and CA-125 criteria every 2 cycles of chemotherapy. Chemotherapy was discontinued with recording of an intolerable toxicity or disease progression. Disease progression in patients prior to 6 cycles (n = 8) of chemotherapy were considered non-responders; and disease progression after 6 cycles (n = 6) of chemotherapy were considered responders. Of the responders, one patient remained without progressive disease for ~17 months. Each patient completed the number of cycles of chemotherapy shown in Fig 2. The responders and non-responders are separated by the red line.

### Circulating miRNAs change in response to chemotherapy

**Fig 3:**
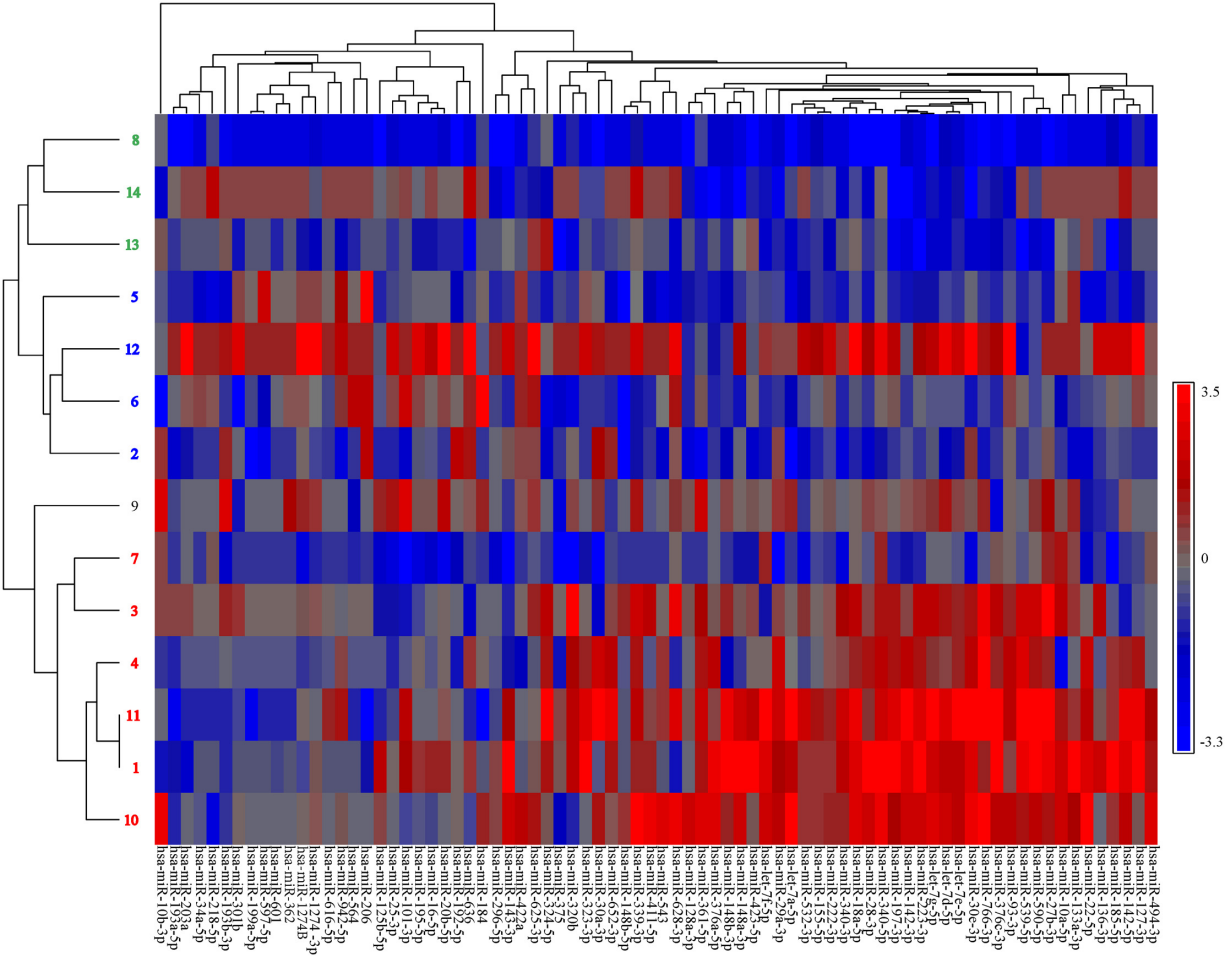
Circulating miRNAs visually differentiate patients into different groups. Patients are numbered (left side of Fig 3) according to convention established, see Fig 2. Patients grouped visually based on clustering (red numbers = group 1, green numbers = group 2, and blue numbers = group 3). The clustering was based on similarity between the changes in miRNA concentrations between baseline (dC_T baseline_) and after chemotherapy treatment (dC_T treatment_). Blue = decreased concentrations after chemotherapy, Red = increased concentrations after treatment.

The primary endpoint of our study was to determine changes in circulating miRNAs following decitabine/carboplatin chemotherapy. Prior to chemotherapy (baseline) and prior to the start of cycle 2 (day 29), plasma concentration were compared for 78 miRNAs. All 78 miRNA were visualized using a Spearman ranked heatmap (Fig 3; same patient identification numbers as in Fig 2). In the heatmap, patients tended to discriminate, after chemotherapy, into groups. Three distinct patient groups were visualized: group1 (patients #1, #3, #4, #7, #10, and #11); group 2 (patients #8, #13, and #14); and group 3 (patients #2, #5, #6, and #12). Groupings were based on the comparison of within patient similarities of the relative changes in miRNA concentrations. For instance, group 3, patient #12 had increased, with treatment, total miRNA concentrations compared to other patients within the group. Patient #12’s miRNAs concentrations changed in the same relative proportions as those recorded for patient #6. Thus, patient #12 and #6 were similarly grouped. As these visualized groupings suggest, this chemotherapy regimen may induce distinct changes in circulating miRNA concentrations in subpopulations.

Next, the changes in the concentrations of individual circulating miRNAs were evaluated after chemotherapy treatment. Following decitabine/carboplatin therapy as shown in Table 1 (individual patient’s miRNA changes shown in S1 Fig), two microRNAs decreased in concentration. miR-193a-5p and miR-375 had respective fold-changes of -1.7 and -2.0 (p<0.05). Additionally, the fold change increased 1.9 (P<0.05) for miR-339-3p.

When the responder subgroup was analyzed, 4 miRNAs, miR-339-3p, miR-340-5p, miR-133a, and miR-10a, increased with a fold range 1.9 to 4.1 (Table 1; S2 Fig). The subgroup of non-responders had three decreasing miRNAs, miR-375, and miR-25-3p, miR-148b-5p. miR-148b-5p had largest decrease (p<0.05) in miRNA concentration (Table 1; S3 Fig).

To determine if individual miRNAs were associated with treatment response, we compared in responders with non-responders the post-treatment concentrations of circulating miRNAs (Fig 2). Three miRNAs, miR-616, and miR-532-3p, miR-148b-5p, were increased in responders (Table 1; S4 Fig). Of these miRNA, miR-148b-5p increase (3.2 fold) in responders was consistent with the subgroup analysis, noted above, revealing its decrease in concentration (2.86) in non-responders.

### Lower miR-148b-5p concentrations predicted worse progression free survival

**Fig 4:**
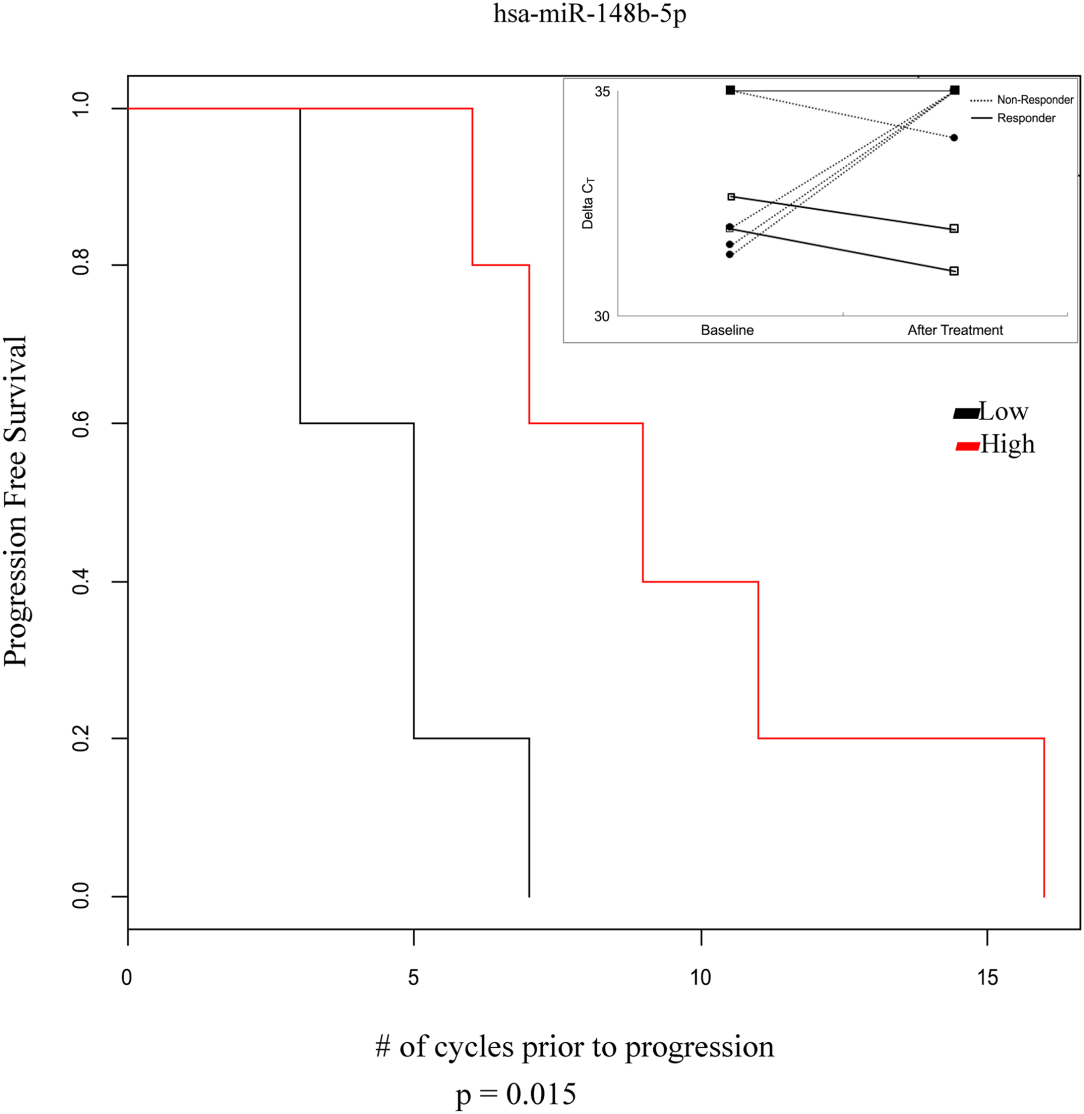
Lower concentrations of miR-148-5p after decitabine/carboplatin chemotherapy compared to baseline predicts worse progression free survival. For the Kaplan Meier progression free survival curve, patient samples were divided into 2 groups by the median value of the “–delta delta CT” values (post treatment value subtracted from baseline value). The log-rank test was between plasma miRNA concentration (high [red line] vs. low [black line]) with the time to non-response, and p value less than 0.05 was considered significant. The inset plots the raw C_T_ values of all patient samples for miR-148-5p, dashed lines—non-responders, solid lines—responders.

Next, a Kaplan Meier PFS analysis was used to evaluate if any of the changes in miRNA concentrations predicted treatment response. To make more applicable to the clinical setting, patients who progressed during the 1st cycle (4 patients) were removed from the analysis. For those patients, progression would be observed using standard diagnostics, and a predictive biomarker would be unnecessary. As shown in Fig 4 of the Kaplan Meier analysis, lower (p = 0.015) miR-148b-5p concentration on day 29 associated with disease progression. Baseline miR-148b-5p concentration was low (Fig 4, inset); and in non-responders, miR-148-5p decreased in concentration largely after 1 month of treatment.

## Discussion

Our study has several important findings. First, decitabine/carboplatin chemotherapy induced changes in circulating miRNA concentrations in patients with platinum-resistant recurrent ovarian cancer. Second, subgroup analysis revealed miRNAs changed distinctly in nonresponders vs. responders. Third, miR-148-5p predicts possible clinical outcome.

Consistently in all patient samples, this chemotherapy regimen induced changes in three circulating miRNAs miR-375, miR-339, and miR-193a. Of these miRNA, miR-193a has been studied previously in human EOC. miR-193a is a tumor suppressor, and overexpression of miR-193a in human EOC cells leads to increased apoptosis [19]. The circulating concentration of miR-193a, in our study, decreased (-1.7-fold) after chemotherapy treatment. The relationship between circulating and tumor miR-193a may warrant further study. Changes in circulating miRNAs common to all patients may be a marker of chemotherapy compliance and dose response.

The observed changes in circulating miRNA concentrations in patients with recurrent ovarian cancer enrolled in this study are similar to previously reported chemotherapy-induced changes in circulating miRNA in patients with melanoma. In that setting, circulating miRNAs predicted drug dose response to dacarbazine and interferon-alfa-2b [20]. miRNA fold changes compared favorably to our study’s -2 to 4 fold changes. The fold changes observed in our study appear reasonable and consistent with those recorded by others.

Subgroup analysis revealed distinctions in miRNA concentrations between non-responders and responders. These distinctions in miRNAs may be a marker of specificity for a subgroup. In the subgroup analysis of non-responders, chemotherapy decreased two additional miRNAs, miR25-3p, and miR148b-5p. Suggesting that these may be specific for a non-responder.

miR-148b-5p predicts possible clinical outcomes. The circulating miR-148b-5p concentration was 3.2 fold higher in our study after decitabine/carboplatin chemotherapy in patients with longer PFS: miR-148b-5p decreased in patients with shorter PFS. Circulating miR-148b-5p has not been reported previously; though, intracellular miR-148-5p has been studied. miR-148b-5p is overexpressed in ovarian cancer tissue; but its overexpression is not associated with histological subtypes [21]. Other studies have shown that changes in intracellular miR-148 (also called miR-148a, and miR-148a-5p) expression, located on chromosome 7, are associated with ovarian cancer outcomes [22–27]. To our knowledge, this is the first study to show that miR-148b, which is located on chromosome 12, is associated with clinical outcomes after treatment with any chemotherapy.

Our study has a couple of strengths. The first strength was a well-described group of patients. This study focused on decitabine/carboplatin chemotherapy induced changes in circulating miRNA focused in recurrent ovarian cancer. Many other studies have been confounded, for example, by either heterogeneous cancer patient population or additional treatments in conjunction with the chemotherapy. For instance, measuring circulating miRNAs as biomarkers for neoadjuvant chemotherapy in breast cancer, one study included surgery and a very heterogeneous population, and the other also included surgery [28, 29]. The results of the studies were not consistent with each other. In the study with chemotherapy followed by surgery, the fold changes in circulating miRNA ranged from 1.5 to 4. In the other study that included patients with the many different stages of breast cancer, the subjects had miRNA fold changes from 1 to 15,000 [28]. Additionally from these studies, circulating miRNA changes induced by chemotherapy were difficult to differentiate from changes caused by surgery. A second strength of our study was the timing of sample collection. Our study samples were collected early in the treatment regimen compared to the studies listed. Our study evaluated changes in circulating miRNA after 1 cycle of chemotherapy; these other studies analyzed changes in circulating miRNAs after 4 cycles of treatment. We propose that evaluating circulating miRNAs after only 1 cycle allows for early clinical decision-making. An important early decision is whether to continue the current chemotherapy regimen or change to another regimen. This decision is especially important for patients with recurrent ovarian cancer, as the progression of disease may be rapid.

A secondary issue is the appropriate utilization of the heatmap. Our heatmap visually identified groups of patients by differences in miRNA concentrations. Though descriptive, the heatmap highlights circulating miRNAs are numerous and potentially altered by chemotherapy. Circulating miRNAs in combination may be a biomarker of chemotherapy response worth exploring in future studies.

We recognized the limitations of our study, including the small sample size. However, despite the small number of patients analyzed, we were still able to detect differences in chemotherapy-induced changes in circulating miRNAs. We acknowledge that these specific miRNA changes may be limited to this regimen, which is not a standard therapy for ovarian cancer patients, and that the patients had previously received a variety of different treatment regimens before starting this trial. Finally, this study included a single time point, a common limitation of these types of studies, and the results suggests that a time course analysis may be warranted in future studies of chemotherapy induced changes in circulating miRNAs.

In conclusion, our study indicates that a carboplatin and decitabine chemotherapy regimen induces changes in circulating miRNAs concentrations that may be predictors of clinical response to this regimen. Our results further indicate that circulating miRNAs may not directly reflect the tumor miRNA expression. These studies indicate that future studies focused on identifying circulating miRNAs predictive of chemotherapy response are warranted.

## Supporting Information

S1 Fig miR-148-5p and miR-25-3p ΔΔ C_T_ changes in patients 1–14. The ΔΔ C_T_ change for the significantly changed miRNAs based on p < 0.05 from the “All subjects treatment/baseline” column in Table 1 are shown for individual patients (listed on x-axis). Red columns–nonresponders (patients 1–8); blue columns–responders (patients 9–14).
(TIF)

S2 Fig miR-340-5p, miR-133a-3p, and miR-10a-5p ΔΔ C_T_ changes in patients 1–14. ΔΔ C_T_ change for the significantly changed from the “Treatment comparison in responsive subjects” column in Table 1 are shown for individual patients (listed on x-axis). miR-339-3p, which significantly changed is already shown in S1 Fig Red columns–nonresponders (patients 1–8); blue columns–responders (patients 9–14).
(TIF)

S3 Fig miR-148b-5p and miR-25-3p ΔΔ C_T_ changes in patients 1–14. ΔΔ C_T_ change for the significantly changed from the “Treatment comparison non-responsive subjects” column in Table 1 are shown for individual patients (listed on x-axis). miR-375 (not shown), which significantly changed is already shown in S1 Fig Red columns–nonresponders (patients 1–8); blue columns–responders (patients 9–14).
(TIF)

S4 Fig miR-616-5p, and miR-532-3p ΔΔ C_T_ changes in patients 1–14. ΔΔ C_T_ change for the significantly changed from the “Treatment comparison in responsive subjects” column in Table 1 are shown for individual patients (listed on x-axis). miR-148b-5p (not shown), which significantly changed is already shown in S2 Fig Red columns–nonresponders (patients 1–8); blue columns–responders (patients 9–14).
(TIF)

S1 File Data used to create Fig 4 and Table 1. (XLSX)

S2 File Data used to create Fig 3 and S1–S4 Figs. (CSV)

S3 File Data used to create inset in Fig 4. (XLSX)

S4 File Data used to create Figs 2 and 4. (XLSX)

## References

[1] Vang R, Shih Ie M, Kurman RJ. Ovarian low-grade and high-grade serous carcinoma: pathogenesis, clinicopathologic and molecular biologic features, and diagnostic problems. Advances in anatomic pathology. 2009;16(5):267–82. 10.1097/PAP.0b013e3181b4fffa 19700937 PMC2745605

[2] Karst AM, Drapkin R. Ovarian cancer pathogenesis: a model in evolution. Journal of oncology. 2010;2010:932371 10.1155/2010/932371 19746182 PMC2739011

[3] Schorge JO, Modesitt SC, Coleman RL, Cohn DE, Kauff ND, Duska LR, et al SGO White Paper on ovarian cancer: etiology, screening and surveillance. Gynecologic oncology. 2010;119(1):7–17. 10.1016/j.ygyno.2010.06.003 20692025

[4] Kjersem JB, Ikdahl T, Lingjaerde OC, Guren T, Tveit KM, Kure EH. Plasma microRNAs predicting clinical outcome in metastatic colorectal cancer patients receiving first-line oxaliplatin-based treatment. Mol Oncol. 2014;8(1):59–67. 10.1016/j.molonc.2013.09.001 24119443 PMC5528512

[5] Esquela-Kerscher A, Slack FJ. Oncomirs—microRNAs with a role in cancer. Nature reviews Cancer. 2006;6(4):259–69. 16557279 10.1038/nrc1840

[6] Mezzanzanica D, Bagnoli M, De Cecco L, Valeri B, Canevari S. Role of microRNAs in ovarian cancer pathogenesis and potential clinical implications. The international journal of biochemistry & cell biology. 2010;42(8):1262–72. 20035894 10.1016/j.biocel.2009.12.017

[7] Lu J, Getz G, Miska EA, Alvarez-Saavedra E, Lamb J, Peck D, et al MicroRNA expression profiles classify human cancers. Nature. 2005;435(7043):834–8. 15944708 10.1038/nature03702

[8] Chen X, Liang H, Zhang J, Zen K, Zhang CY. Horizontal transfer of microRNAs: molecular mechanisms and clinical applications. Protein & cell. 2012;3(1):28–37. 22314808 10.1007/s13238-012-2003-z PMC4875218

[9] Benson EA, Skaar TC. Incubation of whole blood at room temperature does not alter the plasma concentrations of microRNA-16 and -223. Drug metabolism and disposition: the biological fate of chemicals. 2013;41(10):1778–81. 23886700 10.1124/dmd.113.052357 PMC3781369

[10] Mitchell PS, Parkin RK, Kroh EM, Fritz BR, Wyman SK, Pogosova-Agadjanyan EL, et al Circulating microRNAs as stable blood-based markers for cancer detection. Proceedings of the National Academy of Sciences of the United States of America. 2008;105(30):10513–8. 10.1073/pnas.0804549105 18663219 PMC2492472

[11] Zhao YN, Chen GS, Hong SJ. Circulating MicroRNAs in gynecological malignancies: from detection to prediction. Experimental hematology & oncology. 2014;3:14. 24910811 10.1186/2162-3619-3-14 PMC4047546

[12] Zheng H, Zhang L, Zhao Y, Yang D, Song F, Wen Y, et al Plasma miRNAs as diagnostic and prognostic biomarkers for ovarian cancer. PloS one. 2013;8(11):e77853 10.1371/journal.pone.0077853 24223734 PMC3815222

[13] Xiang Y, Ma N, Wang D, Zhang Y, Zhou J, Wu G, et al MiR-152 and miR-185 co-contribute to ovarian cancer cells cisplatin sensitivity by targeting DNMT1 directly: a novel epigenetic therapy independent of decitabine. Oncogene. 2014;33(3):378–86. 10.1038/onc.2012.575 23318422

[14] Zhao HM, Wei W, Sun YH, Gao JH, Wang Q, Zheng JH. MicroRNA-9 promotes tumorigenesis and mediates sensitivity to cisplatin in primary epithelial ovarian cancer cells. Tumour Biol. 2015. 10.1007/s13277-015-3399-x 25846738

[15] Eitan R, Kushnir M, Lithwick-Yanai G, David MB, Hoshen M, Glezerman M, et al Tumor microRNA expression patterns associated with resistance to platinum based chemotherapy and survival in ovarian cancer patients. Gynecologic oncology. 2009;114(2):253–9. 10.1016/j.ygyno.2009.04.024 19446316

[16] Frederick PJ, Green HN, Huang JS, Egger ME, Frieboes HB, Grizzle WE, et al Chemoresistance in ovarian cancer linked to expression of microRNAs. Biotech Histochem. 2013;88(7):403–9. 10.3109/10520295.2013.788736 23672416

[17] Matei D, Fang F, Shen C, Schilder J, Arnold A, Zeng Y, et al Epigenetic resensitization to platinum in ovarian cancer. Cancer research. 2012;72(9):2197–205. 10.1158/0008-5472.CAN-11-3909 22549947 PMC3700422

[18] Plumb JA, Strathdee G, Sludden J, Kaye SB, Brown R. Reversal of drug resistance in human tumor xenografts by 2'-deoxy-5-azacytidine-induced demethylation of the hMLH1 gene promoter. Cancer research. 2000;60(21):6039–44. 11085525

[19] Nakano H, Yamada Y, Miyazawa T, Yoshida T. Gain-of-function microRNA screens identify miR-193a regulating proliferation and apoptosis in epithelial ovarian cancer cells. International journal of oncology. 2013;42(6):1875–82. 10.3892/ijo.2013.1896 23588298 PMC3699598

[20] Triozzi PL, Achberger S, Aldrich W, Singh AD, Grane R, Borden EC. The association of blood angioregulatory microRNA levels with circulating endothelial cells and angiogenic proteins in patients receiving dacarbazine and interferon. Journal of translational medicine. 2012;10:241 10.1186/1479-5876-10-241 23217102 PMC3573971

[21] Chang H, Zhou X, Wang ZN, Song YX, Zhao F, Gao P, et al Increased expression of miR-148b in ovarian carcinoma and its clinical significance. Molecular medicine reports. 2012;5(5):1277–80. 10.3892/mmr.2012.794 22344713

[22] Cancer Genome Atlas Research N. Integrated genomic analyses of ovarian carcinoma. Nature. 2011;474(7353):609–15. 10.1038/nature10166 21720365 PMC3163504

[23] Creighton CJ, Hernandez-Herrera A, Jacobsen A, Levine DA, Mankoo P, Schultz N, et al Integrated analyses of microRNAs demonstrate their widespread influence on gene expression in high-grade serous ovarian carcinoma. PloS one. 2012;7(3):e34546 10.1371/journal.pone.0034546 22479643 PMC3315571

[24] Gu Y, Zhang M, Peng F, Fang L, Zhang Y, Liang H, et al The BRCA1/2-directed miRNA signature predicts a good prognosis in ovarian cancer patients with wild-type BRCA1/2. Oncotarget. 2015;6(4):2397–406. 25537514 10.18632/oncotarget.2963 PMC4385859

[25] Wen Z, Zhao S, Liu S, Liu Y, Li X, Li S. MicroRNA-148a inhibits migration and invasion of ovarian cancer cells via targeting sphingosine-1-phosphate receptor 1. Molecular medicine reports. 2015. 10.3892/mmr.2015.3827 26004261

[26] Zhao S, Wen Z, Liu S, Liu Y, Li X, Ge Y, et al MicroRNA-148a inhibits the proliferation and promotes the paclitaxel-induced apoptosis of ovarian cancer cells by targeting PDIA3. Molecular medicine reports. 2015. 10.3892/mmr.2015.3826 26004124

[27] Zhou X, Zhao F, Wang ZN, Song YX, Chang H, Chiang Y, et al Altered expression of miR-152 and miR-148a in ovarian cancer is related to cell proliferation. Oncology reports. 2012;27(2):447–54. 10.3892/or.2011.1482 21971665

[28] Gezer U, Keskin S, Igci A, Tukenmez M, Tiryakioglu D, Cetinkaya M, et al Abundant circulating microRNAs in breast cancer patients fluctuate considerably during neoadjuvant chemotherapy. Oncology letters. 2014;8(2):845–8. 25009660 10.3892/ol.2014.2188 PMC4081437

[29] Muller V, Gade S, Steinbach B, Loibl S, von Minckwitz G, Untch M, et al Changes in serum levels of miR-21, miR-210, and miR-373 in HER2-positive breast cancer patients undergoing neoadjuvant therapy: a translational research project within the Geparquinto trial. Breast cancer research and treatment. 2014;147(1):61–8. 10.1007/s10549-014-3079-3 25086636

