# Supplementary figures and images for: Carboplatin with Decitabine Therapy, in Recurrent Platinum Resistant Ovarian Cancer, Alters Circulating miRNAs Concentrations: A Pilot Study
Source: PLoS One. 2015 Oct 20;10(10):e0141279. doi: 10.1371/journal.pone.0141279 (PMC4612782; doi:10.1371/journal.pone.0141279)

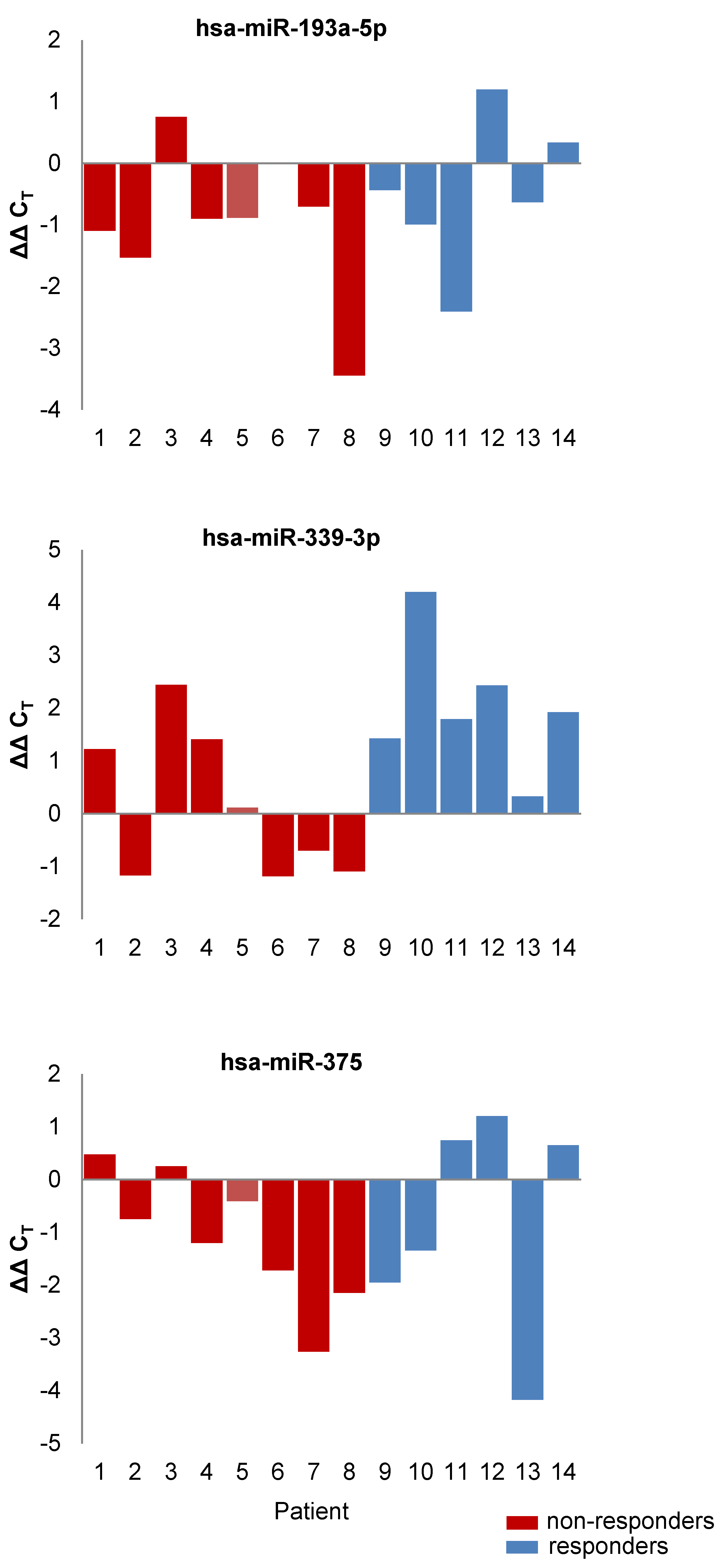

Supplement: S1 Fig — The ΔΔ CT change for the significantly changed miRNAs based on p < 0.05 from the “All subjects treatment/baseline” column in Table 1 are shown for individual patients (listed on x-axis). Red columns–nonresponders (patients 1–8); blue columns–responders (patients 9–14). (TIF) [file pone.0141279.s001.tif]

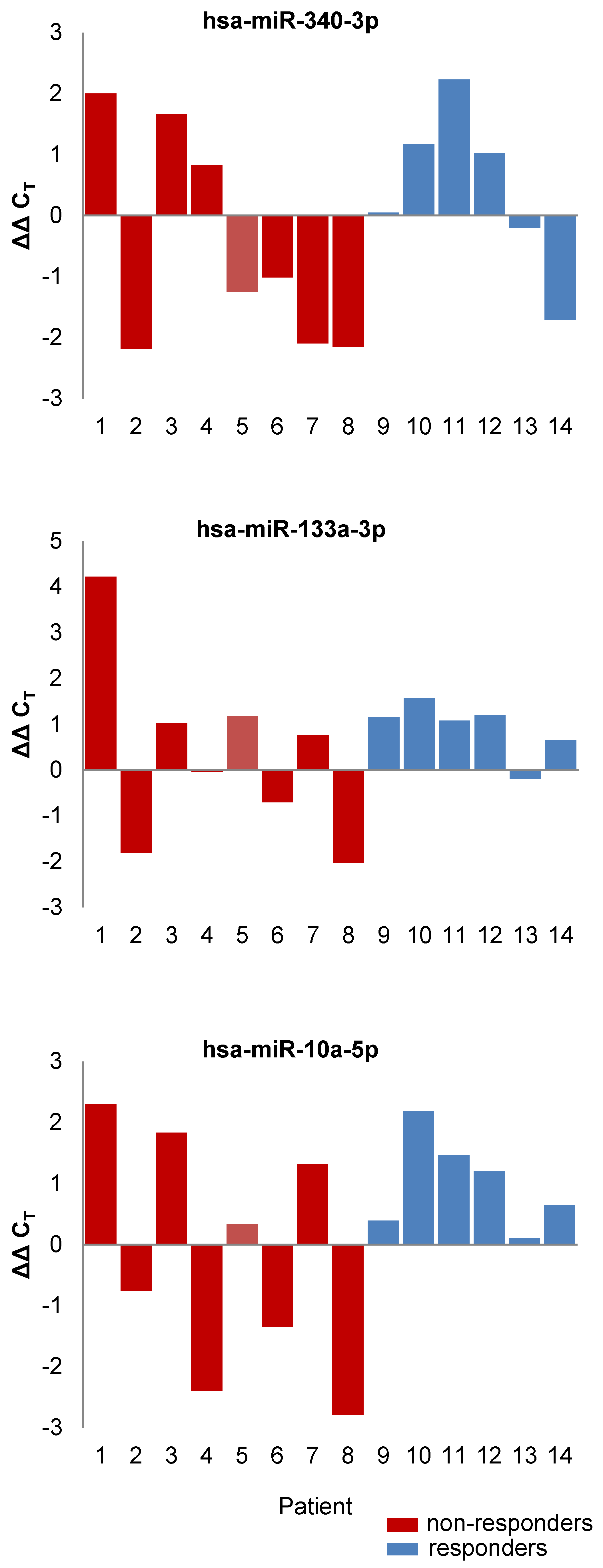

Supplement: S2 Fig — ΔΔ CT change for the significantly changed from the “Treatment comparison in responsive subjects” column in Table 1 are shown for individual patients (listed on x-axis). miR-339-3p, which significantly changed is already shown in S1 Fig Red columns–nonresponders (patients 1–8); blue columns–responders (patients 9–14). (TIF) [file pone.0141279.s002.tif]

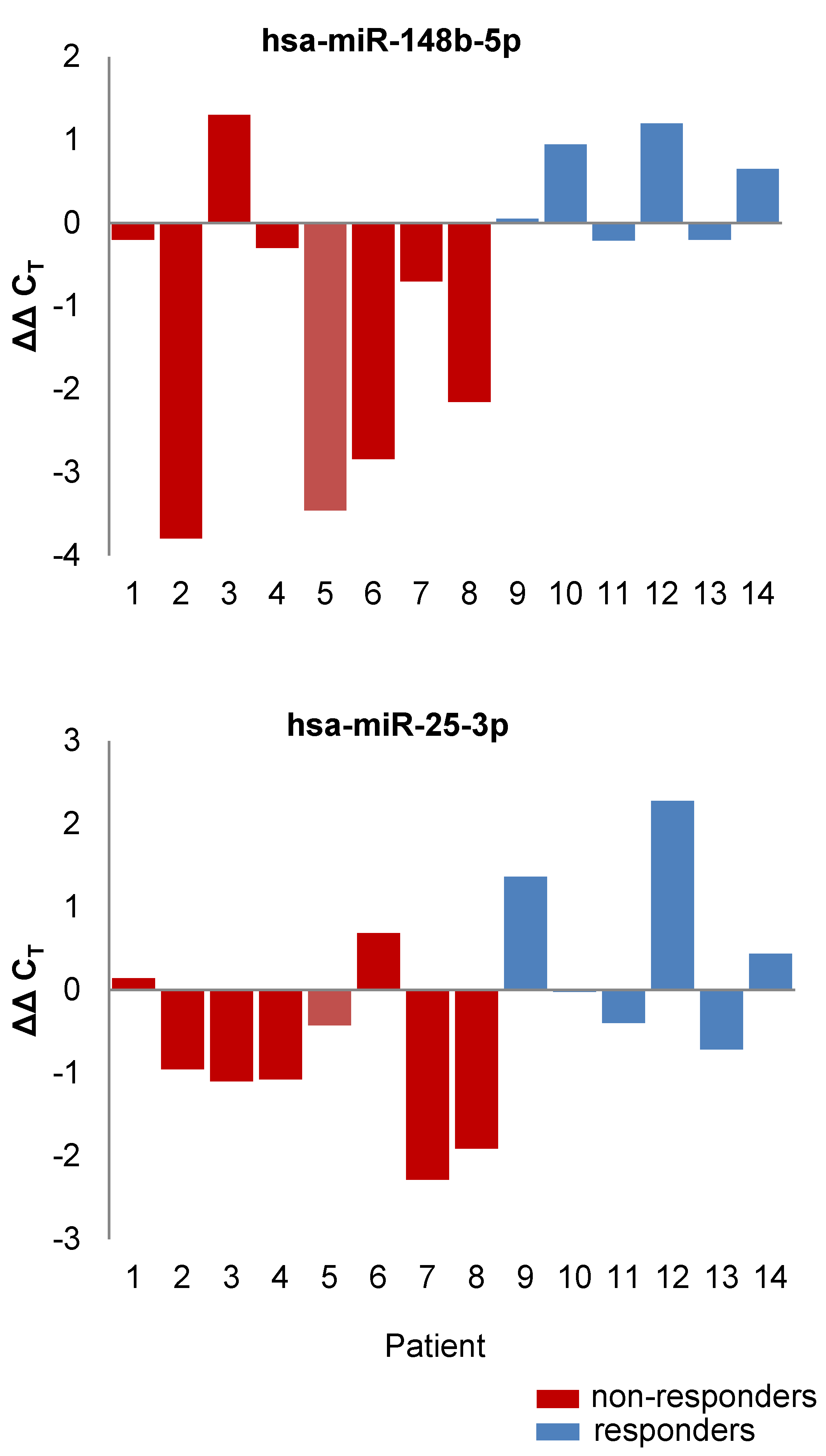

Supplement: S3 Fig — ΔΔ CT change for the significantly changed from the “Treatment comparison non-responsive subjects” column in Table 1 are shown for individual patients (listed on x-axis). miR-375 (not shown), which significantly changed is already shown in S1 Fig Red columns–nonresponders (patients 1–8); blue columns–responders (patients 9–14). (TIF) [file pone.0141279.s003.tif]

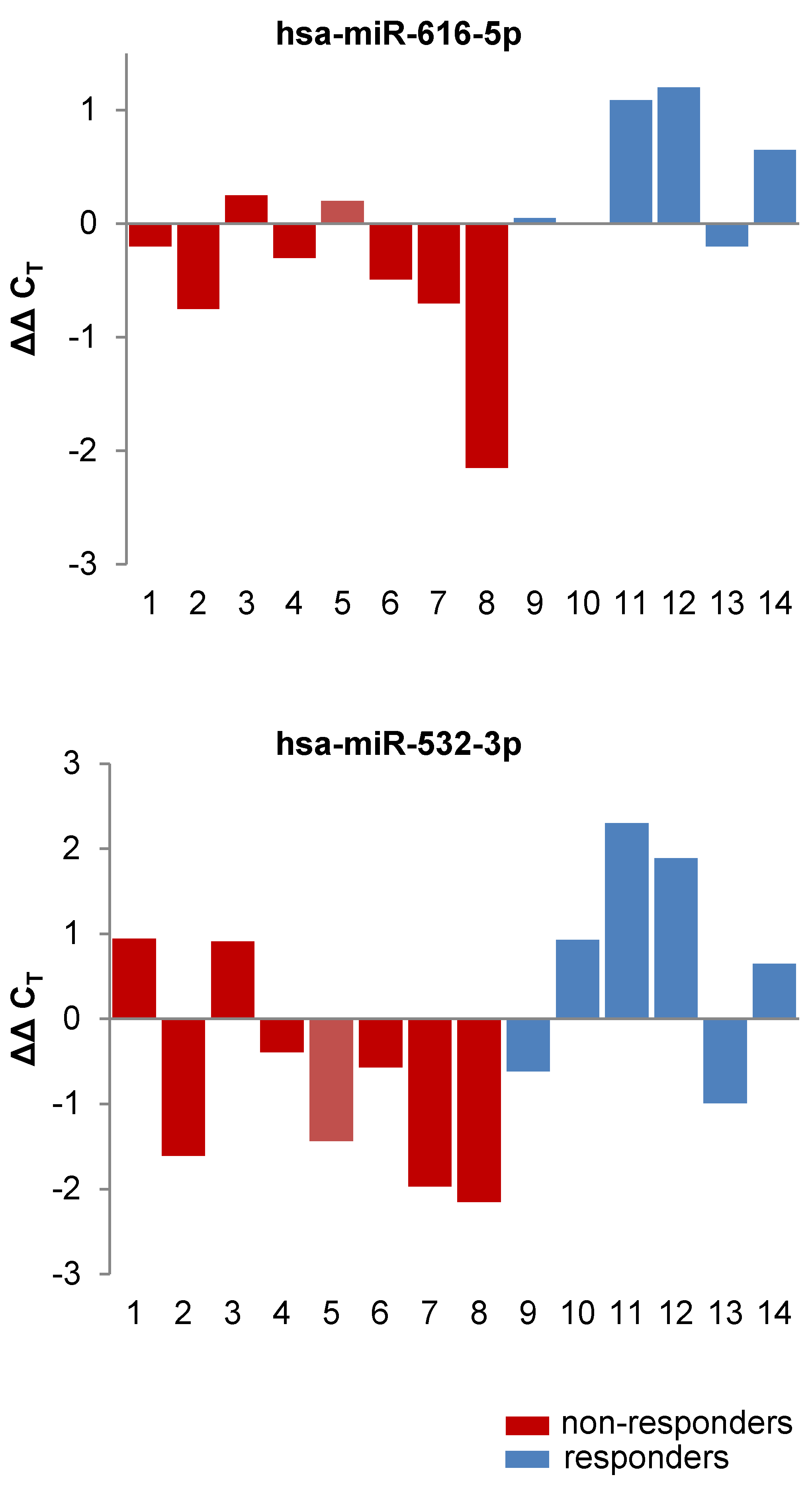

Supplement: S4 Fig — ΔΔ CT change for the significantly changed from the “Treatment comparison in responsive subjects” column in Table 1 are shown for individual patients (listed on x-axis). miR-148b-5p (not shown), which significantly changed is already shown in S2 Fig Red columns–nonresponders (patients 1–8); blue columns–responders (patients 9–14). (TIF) [file pone.0141279.s004.tif]
